# The *Campylobacter jejuni* MarR-like transcriptional regulators RrpA and RrpB both influence bacterial responses to oxidative and aerobic stresses

**DOI:** 10.3389/fmicb.2015.00724

**Published:** 2015-07-21

**Authors:** Ozan Gundogdu, Daiani T. da Silva, Banaz Mohammad, Abdi Elmi, Dominic C. Mills, Brendan W. Wren, Nick Dorrell

**Affiliations:** Faculty of Infectious and Tropical Diseases, London School of Hygiene and Tropical Medicine, London, UK

**Keywords:** *Campylobacter jejuni*, oxidative stress response, aerobic stress response, transcription factors, catalase

## Abstract

The ability of the human intestinal pathogen *Campylobacter jejuni* to respond to oxidative stress is central to bacterial survival both in vivo during infection and in the environment. Re-annotation of the *C. jejuni* NCTC11168 genome revealed the presence of two MarR-type transcriptional regulators Cj1546 and Cj1556, originally annotated as hypothetical proteins, which we have designated RrpA and RrpB (regulator of response to peroxide) respectively. Previously we demonstrated a role for RrpB in both oxidative and aerobic (O_2_) stress and that RrpB was a DNA binding protein with auto-regulatory activity, typical of MarR-type transcriptional regulators. In this study, we show that RrpA is also a DNA binding protein and that a *rrpA* mutant in strain 11168H exhibits increased sensitivity to hydrogen peroxide oxidative stress. Mutation of either *rrpA* or *rrpB* reduces catalase (KatA) expression. However, a *rrpAB* double mutant exhibits higher levels of resistance to hydrogen peroxide oxidative stress, with levels of KatA expression similar to the wild-type strain. Mutation of either *rrpA* or *rrpB* also results in a reduction in the level of *katA* expression, but this reduction was not observed in the *rrpAB* double mutant. Neither the *rrpA* nor *rrpB* mutant exhibits any significant difference in sensitivity to either cumene hydroperoxide or menadione oxidative stresses, but both mutants exhibit a reduced ability to survive aerobic (O_2_) stress, enhanced biofilm formation and reduced virulence in the *Galleria mellonella* infection model. The *rrpAB* double mutant exhibits wild-type levels of biofilm formation and wild-type levels of virulence in the *G mellonella* infection model. Together these data indicate a role for both RrpA and RrpB in the *C. jejuni* peroxide oxidative and aerobic (O_2_) stress responses, enhancing bacterial survival *in vivo* and in the environment.

## Introduction

*Campylobacter* infections are associated with 400 million human cases of gastroenteritis worldwide and *Campylobacter jejuni* is a major cause of bacterial food borne disease and a major causative agent of traveler’s diarrhea (Friedman et al., 2000; Walker, 2005). *C. jejuni* is microaerophilic, growing optimally in an atmosphere of around 10% CO_2_ and 5% O_2_ at a temperature between 37 and 42°C (Park, 2002; Garenaux et al., 2008). However, despite these microaerophilic growth requirements, *C. jejuni* can survive in the ambient environment, which may partly explain the bacteria’s success as a highly prevalent pathogen. Under these less unfavorable conditions *C. jejuni* must have evolved specific adaptation mechanisms (Fields and Thompson, 2008; Olson et al., 2008). *C. jejuni* also encounters oxidative stresses during *in vivo* survival (Fang, 2004; Zaki et al., 2005; Atack and Kelly, 2009; Palyada et al., 2009). Reactive oxygen species (ROS) refers to the chemical species generated upon incomplete reduction of oxygen (Imlay, 2003, 2008). ROS include hydrogen peroxide (H_2_O_2_), the hydroxyl radical (HO^•^) and the superoxide anion (O_2_^–^) (D’Autreaux and Toledano, 2007). The build-up of these toxic compounds in the bacterial cytoplasm and periplasm results in damage to nucleic acids, proteins and membranes. The microaerophilic nature of *C. jejuni* means the bacterium requires a small amount of free oxygen for growth and as such *C. jejuni* will generate ROS in the cytoplasm during metabolism (Sellars et al., 2002; Imlay, 2003). Therefore, the ability to neutralize ROS is essential for *C. jejuni* survival under optimal conditions, in the ambient environment and also within a host where the organism will encounter ROS produced by the host immune system (Imlay, 2008; Atack and Kelly, 2009).

*Campylobacter jejuni* possesses multiple defense mechanisms directly involved in the breakdown of ROS. Catalase converts H_2_O_2_ to H_2_O and O_2_ and *C. jejuni* contains the catalase KatA which is induced by both H_2_O_2_ and O_2_^–^ (Grant and Park, 1995; Garenaux et al., 2009). The superoxide dismutase SodB converts O_2_^–^ to H_2_O_2_ and O_2_ (Hassan, 1988). Studies have demonstrated that *C. jejuni* NCTC11168 expresses KatA, but not SodB, when exposed to O_2_^–^ (Garenaux et al., 2009). However, a basal level of SodB expression has been shown to be important as *sodB* mutants are overly sensitive to oxidative stress (Pesci et al., 1994). *C. jejuni* contains a number of different types of peroxiredoxins, cellular antioxidant proteins which control intracellular peroxide levels (Hall et al., 2009). These non-haem proteins have been shown to confer resistance to H_2_O_2_ and organic hydroperoxides through breakdown to H_2_O and the corresponding alcohol (Wood et al., 2003). *C. jejuni* possesses the alkyl hydroperoxide reductase AhpC (Baillon et al., 1999; Poole et al., 2000), the thiol peroxidase Tpx (Atack et al., 2008) and the bacterioferritin co-migratory protein Bcp (Atack et al., 2008).

However, all *C. jejuni* strains lack some of the classical bacterial stress response regulatory proteins found in other enteric bacteria such as the SoxRS and OxyR regulons (Pomposiello et al., 2001; Blanchard et al., 2007; Imlay, 2008). In *C. jejuni* NCTC11168 the peroxide sensing regulator PerR was demonstrated to repress *katA* and *ahpC* transcription in an iron-dependent manner, indicating that PerR acts as a functional, but non-homologous substitute for OxyR (van Vliet et al., 1999). All *C. jejuni* strains contain the ferric uptake regulator (Fur) which is an iron-responsive regulator controlling the expression of genes encoding iron uptake systems (Stintzi et al., 2008). The control of iron metabolism and the response to oxidative stress are closely linked, as ROS are generated by the combination of iron with oxygen (van Vliet et al., 2002). Fur directly regulates expression of *katA*, as both *fur* and *perR* must be mutated to completely abolish iron-dependent *katA* expression (van Vliet et al., 1999; Palyada et al., 2009). *C. jejuni* also contains the *Campylobacter* oxidative stress regulator CosR, which was shown to be responsible for the negative regulation of the oxidative stress response proteins SodB, Dps, Rrc, and LuxS and positive regulation of AhpC and KatA (Hwang et al., 2011, 2012). *C. jejuni* CsrA has been identified to be a post-transcriptional regulator and a *C. jejuni* 81–176 *csrA* mutant displayed increased sensitivity to various stresses including atmospheric oxygen and H_2_O_2_ (Fields and Thompson, 2008). *C. jejuni* CprRS has been shown to be important for oxidative stress resistance (Svensson et al., 2009). The response regulator CprR appears to be essential as mutations in *cprR* were lethal, however, the sensor kinase CprS is inessential and a 81–176 *cprS* mutant exhibited decreased oxidative stress tolerance to t-butylhydroperoxide, paraquat and H_2_O_2_ (Svensson et al., 2009).

Cj1556 was identified as a MarR-type transcriptional regulator during the re-annotation and re-analyze of the *C. jejuni* NCTC11168 genome sequence and further investigation demonstrated a role for Cj1556 in the responses to both oxidative and aerobic (O_2_) stress (Gundogdu et al., 2007, 2011). Mutation of *Cj1556* in the 11168H strain increased sensitivity to oxidative and aerobic (O_2_) stress. The 11168H *Cj1556* mutant exhibited a decrease in virulence in the *Galleria mellonella* infection model and a reduced ability for intracellular survival in Caco-2 human intestinal epithelial cells and J774A.1 mouse macrophages. Negative autoregulation of *Cj1556* expression was identified using microarray analysis of gene expression changes in the *Cj1556* mutant and also down-regulation of both *katA* and *perR*. The binding of recombinant Cj1556 to the promoter region upstream of the *Cj1556* gene was confirmed by electrophoretic mobility shift assays (EMSAs). Further analysis of the re-annotated NCTC11168 genome identified a second MarR transcriptional regulator Cj1546 located in close proximity to Cj1556 on the NCTC11168 chromosome (Gundogdu et al., 2007, 2011). In this study, we show that in the 11168H wild-type strain, Cj1546 also plays a role in regulating the *C. jejuni* oxidative and aerobic (O_2_) stress responses and specifically is involved in responding to peroxide stress. We have thus respectively designated Cj1546 and Cj1556 as RrpA and RrpB (regulator of response to peroxide).

## Materials and Methods

### Bacterial Strains and Growth Conditions

All *C. jejuni* strains used in this study are listed in Table 1. *C. jejuni* strains were grown in either *Brucella* or Mueller Hinton broth (Oxoid) with shaking at 75 rpm or on blood agar (BA) plates containing Columbia agar base (Oxoid, UK) supplemented with 7% (v/v) horse blood (TCS Microbiology, UK) and *Campylobacter* Selective Supplement (Oxoid). *C. jejuni* strains were grown at 37°C in a microaerobic chamber (Don Whitley Scientific, UK), containing 85% N_2_, 10% CO_2_, and 5% O_2_. *C. jejuni* strains were grown on BA plates for 24 h prior to use in assays and co-culture experiments. *Escherichia coli* XL-2 Blue MRF’ competent cells (Stratagene, UK) were used for cloning experiments and were grown at 37°C in aerobic conditions either in LB broth (Oxoid) with shaking at 200 rpm or on Luria-Bertani (LB) agar plates (Oxoid). Antibiotics were added at the following concentrations; kanamycin (50 μg/ml), ampicillin (100 μg/ml), and chloramphenicol (50 μg/ml for *E. coli* studies or 10 μg/ml for *C. jejuni* studies). All reagents were obtained from Fisher Scientific (UK) unless otherwise stated.

**TABLE 1:** **Bacterial strains used in this study**.

**Campylobacter jejuni strains**	**Description**	**References**
11168H	A hypermotile derivative of the original sequence strain NCTC11168 that shows higher levels of caecal colonization in a chick colonization model.	Karlyshev et al. (2002); Jones et al. (2004)
11168H *rrpA* mutant	Isogenic 11168H *rrpA* mutant with insertion of a 1.4 kb Kan^R^ cassette.	This study
11168H *rrpA* complement	*rrpA* complement constructed by the insertion of the *rrpA* CDS into a rRNA gene in the 11168H *rrpA* mutant (pRRC complementation vector used).	This study
11168H *rrpA* complement-HIS	*rrpA* 6 His tagged complement constructed by the insertion of the *rrpA* CDS into a rRNA gene in the 11168H *rrpA* mutant (pRRC complementation vector used).	This study
XL1 pEXT20 *rrpA* HIS	*rrpA* with a 6 His tag cloned into pEXT20 cloned in *E. coli* XL1 Blue strain allowing recombinant RrpA to be isolated.	This study
11168H *rrpAB* mutant	11168H double mutant strain containing a 1.4 kb Kan^R^ cassette within *rrpA* and 0.8 kb Cat^R^ cassette within *rrpB*.	This study
11168H *perR* mutant	Obtained from *Campylobacter* mutant bank http://crf.lshtm.ac.uk/wren_mutants.htm	LSHTM mutant bank
11168H *sodB* mutant	Obtained from *Campylobacter* mutant bank http://crf.lshtm.ac.uk/wren_mutants.htm	LSHTM mutant bank
11168H *ahpC* mutant	Obtained from *Campylobacter* mutant bank http://crf.lshtm.ac.uk/wren_mutants.htm	LSHTM mutant bank
11168H *katA* mutant	Obtained from *Campylobacter* mutant bank http://crf.lshtm.ac.uk/wren_mutants.htm	LSHTM mutant bank

### Construction of *C. jejuni* Mutants and Complements

*Campylobacter jejuni* mutants were constructed as described previously (Gundogdu et al., 2011). Briefly, genes or gene fragments were amplified from *C. jejuni* genomic DNA using the appropriate gene specific primers as listed in Table 2. PCR products were ligated with pGEM-T Easy vector (Promega, UK) and then transformed into XL-2 Blue MRF’ cells. If required, inverse PCR mutagenesis (IPCRM) was performed to introduce a unique BglII site into the cloned gene. A kanamycin cassette (Kan^R^) was then ligated into the unique BglII site within the cloned gene (Trieu-Cuot et al., 1985; van Vliet et al., 1998). These constructs were electroporated into competent *C. jejuni* cells and putative clones were confirmed by PCR and sequencing as described previously (Gundogdu et al., 2011). The same procedure was used for construction of double mutants, except a chloramphenicol cassette (Cat^R^) was used to introduce the second mutation (BamHI restriction site). Complementation of mutant strains was performed as described previously (Gundogdu et al., 2011). Briefly, the complete gene sequence plus the native ribosome binding site was amplified by PCR and cloned into pRRC complementation vector (Karlyshev and Wren, 2005). The complemented mutant was constructed both with (11168H *rrpA* complement-HIS) and without (11168H *rrpA* complement) a 6 × His tag to resuscitate observed phenotypes and to ensure no additional effect was caused by the introduction of the 6 × His tag. All constructs were confirmed with PCR and sequencing. For isolation of recombinant RrpA protein, a 6 × His tag sequence was cloned into pEXT20 (Dykxhoorn et al., 1996) using primers *rrpA*-HIS-pEXT20-F and *rrpA*-HIS-pEXT20-R with the optimized RBS sequence AGGAGGTAAAACAT (XL1 pEXT20 *rrpA* HIS). The native host strain for complementation constructs using pRRC and pEXT20 was XL-1 Blue subcloning competent cells (Life Technologies, USA).

**TABLE 2:** **Oligonucleotide primers used in this study**.

**Primer Name**	**Sequence**
*rrpA*-F	TACTAGGATTTTCATGAG
*rrpA*-R	AGATGTTAAATCTCACTGCT
*rrpA*-IPCRF	GGGAGATCTCTCTTAAGGTATTGGTTA
*rrpA*-IPCRR	GGGAGATCTCGATGGTTTAATTATCAG
Km^R^ forward-out	TGGGTTTCAAGCATTAGTCCATGCAAG
Km^R^ reverse-out	GTGGTATGACATTGCCTTCTGCG
Cat forward-out	CGATTGATGATCGTTGTA
Cat reverse-out	TACAGCAGACTATACTG
Comp-*rrpA*-F	CCCTCTAGACTAAAGGAATGTTAAATGACTAAA GAGAATTCTCCG
Comp-*rrpA*-R	CCCTCTAGATTAATTCAAGCATTTTTTCCC
Comp-*rrpA*-R-HIS	CCCTCTAGATTAATGATGATGATGATGATGATT CAAGCATTTTTTCCC
*rrpA*-HIS-pEXT20-F	CCCGAGCTCAGGAGGTAAAACATATGACTAAA GAGAATTCTCCG
*rrpA*-HIS-pEXT20-R	CCCTCTAGATTAATGATGATGATGATGATGATTC AAGCATTTTTTCCC
pEXT20 forward	AGCGGATAACAATTTCACAC
pEXT20 reverse	CGACGAATTTCTTCTCTCAT
*katA* RT-F	TGTCCTGAAAGTTTACATC
*katA* RT-R	CATAGCACCAGCGACATTG
*gyrA* RT-F	CGACTTACACGGCCGATTTC
*gyrA* RT-R	ATGCTCTTTGCAGTAACCAAAAAA
Upstream *rrpA*-F	TTAGCACCATTAAGCAAG
Upstream *rrpA*-R	GTTAAATCCACATTCTTCG
Upstream *flaA*-F	ATCACAGCTTATATTAAAG
Upstream *flaA*-R	GTGTTAATACGAAATCCCAT
Upstream *katA*-F	ATCTGCACCAATAACC
Upstream *katA*-R	GAATTTTGGTTATCAGCTA

### Electrophoretic Mobility Shift assays

To obtain recombinant RrpA, XL1 pEXT20 *rrpA* HIS was grown at 37°C for 16 h with shaking at 200 rpm, then centrifuged at 4,000 rpm at 4°C for 10 min. Bacterial pellets were resuspended in 1 ml equilibration buffer (Sigma-Aldrich, UK), sonicated with a Diagenode Bioruptor using manufacturers instructions (Diagenode, Belgium), then centrifuged for 5 min at 13,000 rpm. The supernatant was transferred into a fresh 1.5 ml tube. Lysed cells were mixed with Ni-NTA (Qiagen, Netherlands) then incubated at 4°C for 1 h on a rotator. Elution was performed using a His-Select spin column (Sigma-Aldrich) and stored under native conditions. An aliquot of the elution was separated and denatured using an equal volume of 2X Laemmli buffer (Sigma-Aldrich), followed by boiling the samples for 10 min then centrifuged at 13,000 rpm for 5 min. Denatured samples were assessed on a 12% NuPAGE® Bis-Tris (Novex) gels (Life Technologies). In addition, the original native aliquot was assessed for concentration using Pierce^™^ BCA Protein Assay Kit (Life Technologies). To demonstrate the DNA binding properties of RrpA, purified recombinant protein was hybridized to IRDye® 700 PCR amplified fragment (160 bp) located upstream of the translation initiation sites of the *rrpA* or *katA* gene (Tables 2 and 3). 2.5 μg (≈175 pmol) recombinant native protein was hybridized with 20 ng of gel purified DNA using the Odyssey® Infrared EMSA kit (LI-COR Biosciences, USA). Unlabelled *rrpA*/*katA*/*flaA* upstream fragments were used as controls. Samples were loaded in a pre-cast 6% Novex® DNA retardation gel (Life Technologies) and run at 4°C. Samples were analyzed on a LI-COR Odyssey® imaging scanner (LI-COR Biosciences).

**TABLE 3:** **DNA fragments used as promoter probes for electrophoretic mobility shift assays**.

**Fragment region**	**Purpose of selection**	**Location within genome (nucleotides)**	**Size of fragment (bp)**
Upstream of *rrpA*	Proposed binding site	1477180 – 1477340	160
Upstream of *flaA*	Negative control	1271120 – 1270940	180
Upstream of *katA*	Proposed binding site	1322330 – 1322560	230

### Oxidative Stress and Aerobic Growth Assays

Bacterial cells were harvested into 1 ml PBS and diluted to an OD_600_ of 1. For oxidative stress assays, bacterial cells were exposed to H_2_O_2_ at final concentrations of 25, 50, or 100 mM for 15 min, menadione at a final concentration of 100 mM for 60 min and cumene hydroperoxide at 0.05% (w/v) for 15 min, all at 37°C under microaerobic conditions. Serial dilutions were prepared and 10 μl of the 10^–1^ – 10^–6^ dilutions were spotted onto BA plates, incubated for 48 h and colonies counted. For growth curves, 10 ml Brucella broth was pre-incubated in a 30 ml flask at 37°C under microaerobic conditions for 24 h. Bacterial cells grown on BA plates for 24 h were used to inoculate pre-incubated Brucella broth at an OD_600_ of 0.1 and grown for up to 24 h at 37°C under microaerobic and aerobic conditions. OD_600_ readings were performed at selected time points. In addition bacterial colony forming units (CFUs) were assessed at time point 16 h under microaerobic and aerobic conditions.

### Catalase Activity Assays

*Campylobacter jejuni* cells were harvested and resuspended in 1 ml PBS, sonicated three times at 60 kHz for 30 s on ice, then centrifuged at 13,000 rpm for 15 min at 4°C. Supernatants were removed and stored on ice. A BCA Protein Assay Kit (Thermo Scientific, USA) was used to quantify the protein concentration of each supernatant, which were adjusted to a final concentration of 100 μg/ml with PBS. The catalase activity of each supernatant was quantified using the Catalase Assay Kit (Sigma-Aldrich). Each catalase activity assay was performed on 1 μg total protein and the decomposition of H_2_O_2_ was measured after 1 min. A unit of catalase activity is defined as 1 μmol H_2_O_2_ decomposed per min at 25°C.

#### RT-PCR

*Campylobacter jejuni* RNA was isolated from 16 h cultures using the RNeasy Mini purification kit (Qiagen) and RNA*later* RNA Stabilization Reagent (Qiagen) as described previously (Kamal et al., 2007). cDNA was synthesized by reverse transcription using SuperScript® III First-Strand Synthesis System (Invitrogen, USA). Primers *katA* RT-F and *katA* RT-R were used to amplify *katA*. Primers *gyrA* RT-F and *gyrA* RT-R were used to amplify the endogenous control *gyrA*. The PCR products were resolved by electrophoresis on a 0.7% (w/v) agarose gel and recorded by a fluorescence scanner (GeneGenius, Syngene, UK). Band volumes were quantified with ImageJ software (http://imagej.nih.gov/ij/) as described previously (Schneider et al., 2012). *katA* cDNA PCR product ratios were calculated and normalized to *gyrA*. Gene expression levels are described as relative intensity (vs. *gyrA*).

### Biofilm Assays

Bacterial cells were harvested into Mueller Hinton broth, then inoculated to an OD_600_ of 0.1 into 10 ml Mueller Hinton broth pre-incubated in a 25 ml flask at 37°C under microaerobic conditions for 24 h prior to inoculation then grown for 5 h under microaerobic conditions with shaking at 75 rpm. The OD_600_ was readjusted to 0.1, then 1 ml of culture was added to a 24 well polystyrene plates (Corning, USA) and incubated at 37°C under either aerobic or microaerobic conditions stationary for 72 h. The wells were washed two times with PBS, dried for 20 min at 37°C followed by addition of 1% (w/v) crystal violet (Sigma-Aldrich) for 15 min. The wells were washed three times with PBS, then destained with 10% (v/v) acetic acid / 30% (v/v) methanol. Absorbance (*A*_595_) was measured using a SpectraMax M3 microplate reader (Molecular Devices, USA).

### *Galleria mellonella* Infection Model

*Galleria mellonella* larvae (LiveFoods Direct, UK) were stored at 16°C on wood chips. 10 larvae for each experiment were infected with a 10 μl inoculum of a 24 h *C. jejuni* culture diluted to OD_600_ 0.1 by micro-injection (Hamilton, Switzerland) in the right foremost leg, giving an infectious dose of approximately 10^6^ CFU (Champion et al., 2010). Controls were injection with PBS and no injection. Larvae were incubated at 37°C with survival recorded at 24 h.

### Statistical Analyses

The data is presented as mean + SD. All experiments were performed with at least three biological replicates, performed in triplicate in each experiment. Statistical analyses were performed using Prism software (GraphPad Software). Variables were compared using a student’s *t*-test.

## Results

### Electrophoretic Mobility Shift Assays Indicate RrpA is also a DNA Binding Protein

The re-annotation of *C. jejuni* NCTC11168 genome sequence (Gundogdu et al., 2007) identified two MarR–type transcriptional regulators (Cj1546 and Cj1556) based on the presence of a Pfam motif (PF01638). PF01638 is a HxlR-like helix-turn-helix motif and a member of the MarR family of transcriptional regulators that regulate virulence factor expression, bacterial responses to both antibiotics and oxidative stress, as well as catabolism of environmental aromatics compounds (Wilkinson and Grove, 2004; Wösten et al., 2008). We have designated Cj1546 as RrpA (regulator of response to peroxide) and Cj1556 as RrpB. RrpA has 43.6% identity and 58.4% similarity to RrpB.

**FIGURE 1:**
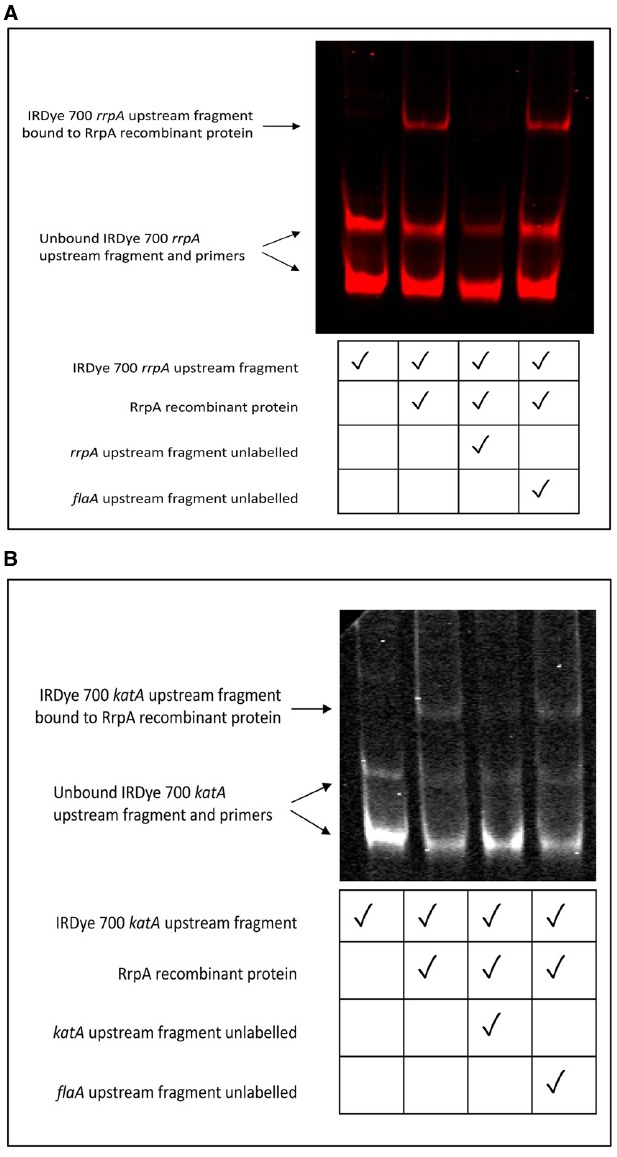
**Electrophoretic mobility shift assays (EMSAs) indicate that RrpA binds to a DNA promoter sequence upstream of the rrpA (A) and *katA* gene (B).** EMSAs were performed for RrpA protein hybridized to a PCR amplified fragment upstream of *rrpA* or *katA* labeled with IRDye® 700. 2.5 μg (≈175 pmol) recombinant native protein was hybridized with 20 ng of gel purified DNA using the Odyssey® Infrared EMSA kit (LI-COR Biosciences). Unlabelled PCR amplified fragments upstream of either *rrpA*, *katA*, or *flaA* were included as controls. Separation was performed under non-denaturing conditions with samples loaded onto a 6% (w/v) DNA retardation gel.

RrpB is a DNA binding protein that binds to the promoter region of the *rrpB* gene resulting in negative autoregulation, a feature of the MarR family of transcriptional regulators (Gundogdu et al., 2011). EMSAs were performed to investigate whether RrpA could bind to the promoter region of the *rrpA* gene. The full-length RrpA protein was expressed and purified from *E. coli*. We do not believe the 6 × His interfered with any observed phenotypes as we have constructed the *rrpA* 6 × His tagged in the complementation vector pRRC as a control and obtained resuscitation of the hydrogen peroxide phenotype. This recombinant RrpA protein was observed to bind to a IRDye 700 fluorescently labeled 160 bp DNA fragment upstream of the *rrpA* gene (Figure 1A). Unlabelled upstream regions of *rrpA* and *flaA* were used as controls. An excess of unlabelled *rrpA* upstream region competed with the IRDye® 700 fluorescently labeled *rrpA* upstream region for the RrpA recombinant protein. However, an excess of unlabelled *flaA* upstream region did not compete with the RrpA recombinant protein. This data indicates that RrpA acts as a DNA binding protein. MarR family transcriptional regulators are classically negative autoregulators, so whilst RrpA and RrpB have been shown to bind upstream of *rrpA* and *rrpB* respectively, it is not possible to infer whether this is negative or positive autoregulation based on this experimental data. In addition, RrpA was shown to bind upstream of *katA* (Figure 1B).

### 11168H *rrpA* and *rrpB* Mutants Exhibit Increased Sensitivity to Hydrogen Peroxide Stress and Reduced Catalase Activity

**FIGURE 2:**
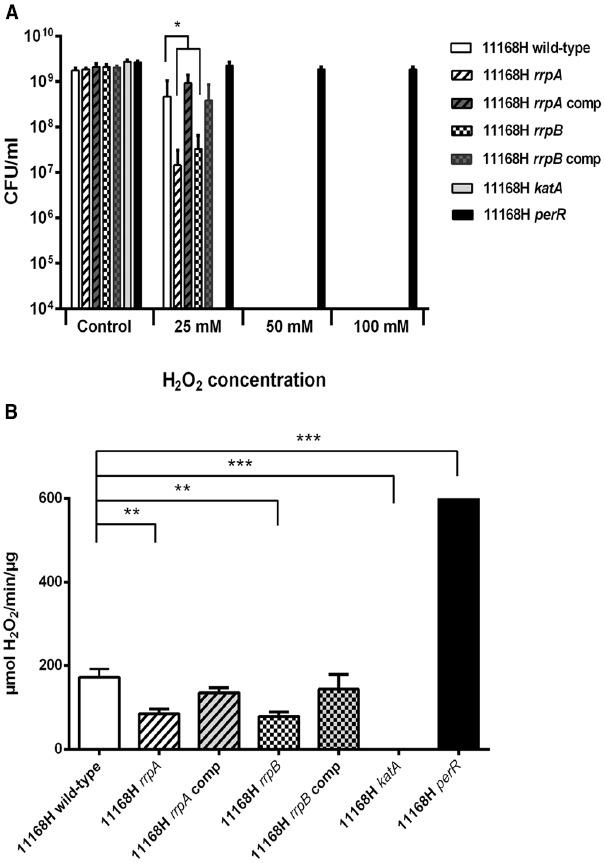
**(A)** Effect of oxidative stress on the survival of *C. jejuni* 11168H wild-type strain, *rrpA*, *rrpA* complement strain (*rrpA* comp), *rrpB*, *rrpB* complement strain (*rrpB* comp), *katA* and *perR* mutants. *C. jejuni* strains were incubated with 25, 50, or 100 mM H_2_O_2_ for 15 min at 37°C then bacterial survival assessed. **(B)** Catalase activity assays were performed on bacterial whole cell lysates from the 11168H wild-type strain, *rrpA*, *rrpA* comp, *rrpB*, *rrpB* comp, *katA* and *perR* mutants for 1 min. Asterisks denote a statistically significant difference (*p < 0.05, **p < 0.01, ***p < 0.001).

The *rrpA* mutant and *rrpA* complement were tested for sensitivity to H_2_O_2_ (25, 50, and 100 mM). The *rrpA* mutant exhibited increased sensitivity to 25 mM H_2_O_2_ stress, whilst the *rrpA* complement exhibited survival similar to the wild-type strain (Figure 2A). As controls, H_2_O_2_ stress assays were also performed on 11168H *katA* and *perR* mutants. The *perR* mutant displayed a high level of resistance to H_2_O_2_ stress, in confirmation of a previous study which demonstrated that a *C. jejuni* NCTC11168 *perR* mutant constitutively expressed both *katA* and *ahpC*, resulting in enhanced resistance to peroxide stress compared to the wild-type strain (van Vliet et al., 1999). The *katA* mutant does not express catalase and did not survive exposure to any concentration of H_2_O_2_. Catalase activity assays were performed on bacterial whole cell lysates. Both the *rrpA* and *rrpB* mutants exhibited reduced catalase activity compared to the wild-type strain, whilst the *katA* mutant had no catalase activity and the *perR* mutant exhibited significantly increased catalase activity (Figure 2B). Catalase activity was restored to wild-type levels in both the *rrpA* and *rrpB* complements (Figure 2).

### 11168H *rrpAB* Double Mutant Exhibits Reduced Sensitivity to Hydrogen Peroxide Stress

**FIGURE 3:**
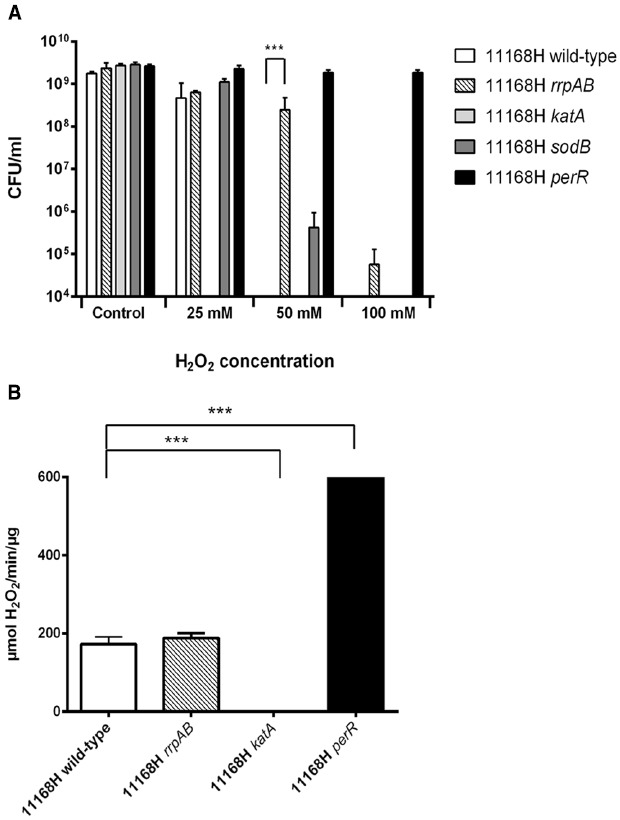
**(A)** Effect of oxidative stress on the survival of *C. jejuni* 11168H wild-type strain and *rrpAB*, *katA*, and *perR* mutants. *C. jejuni* strains were incubated with 25, 50, or 100 mM H_2_O_2_ for 15 min at 37°C and bacterial survival assessed. **(B)** Catalase activity assays were performed on bacterial whole cell lysates from the 11168H wild-type strain and *rrpAB*, *katA* and *perR* mutants for 1 min. Asterisks denote a statistically significant difference (***p < 0.001).

To further study the role of both RrpA and RrpB in the *C. jejuni* oxidative stress response, the 11168H *rrpAB* mutant was tested for sensitivity to H_2_O_2_. The 11168H *rrpAB* mutant exhibited increased resistance to 25 mM H_2_O_2_ compared to even the wild-type strain (Figure 3A). The *rrpAB* mutant also survived exposure to higher concentrations of H_2_O_2_ (50 or 100 mM), but exhibited levels of catalase activity similar to the wild-type strain (Figure 3B).

### 11168H *rrpA* and *rrpB* Mutants Exhibit Reduced *katA* Transcription Levels

**FIGURE 4:**
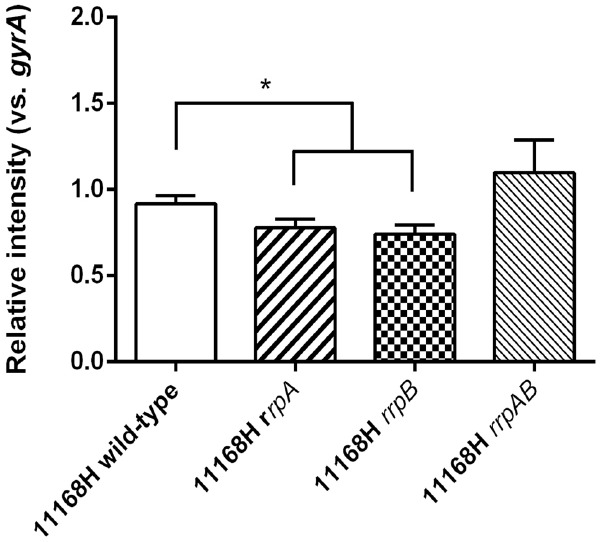
**RT-PCR analysis of *katA* transcription in *C. jejuni* 11168H wild-type strain and *rrpA*,*rrpB*, and *rrpAB* mutants.** *C. jejuni* RNA was converted to cDNA and semi-quantitative levels of *katA* expression were measured as relative intensity to the internal control *gyrA*. Asterisks denote a statistically significant difference (*p < 0.05).

Semi-quantitative RT-PCR was performed investigating the expression levels of *katA* in the *rrpA*, *rrpB*, and *rrpAB* mutants compared to the 11168H wild-type strain (Figure 4). The amount of expression was calculated using the endogenous control *gyrA* and presented as relative intensity (vs. *gyrA*). The *rrpA* and *rrpB* mutants exhibited a significant reduction in the level of *katA* production when compared to the 11168H wild-type strain. In contrast, the *rrpAB* mutant displayed increased levels of *katA* expression compared to the 11168H wild-type strain, however, this increase was not significant.

### Mutation of *rrpA* and *rrpB* Does not Increase Sensitivity to Cumene Hydroperoxide or Menadione Stress

**FIGURE 5:**
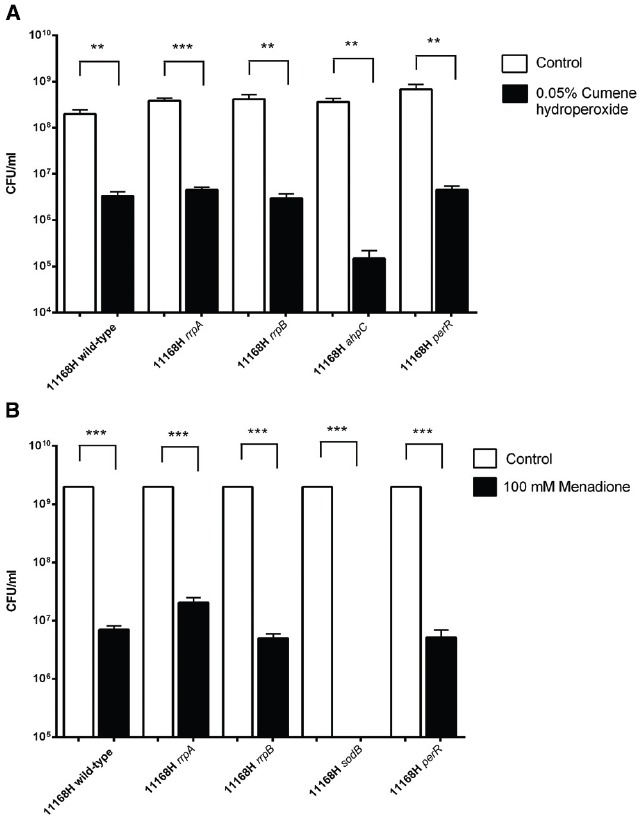
**Effect of (A) cumene hydroperoxide and (B) menadione on the survival of *C. jejuni* 11168H wild-type strain or mutants.** Strains were exposed to 0.05% (w/v) cumene hydroperoxide for 15 min or 100 mM menadione for 1 h at 37°C under microaerobic conditions then bacterial survival assessed. Asterisks denote a statistically significant difference (**p < 0.01, ***p < 0.001).

To investigate the role of RrpA and RrpB in the response to different oxidative stresses, the *rrpA* and *rrpB* mutants were also tested for sensitivity to cumene hydroperoxide (an organic hydroperoxide) and menadione (a superoxide generating agent). Neither the *rrpA* nor *rrpB* mutants exhibited any change in sensitivity to either cumene hydroperoxide (Figure 5A) or menadione (Figure 5B) stress compared to the wild-type strain. A *ahpC* mutant was used as a control for cumene hydroperoxide stress as AhpC is known to break down this compound (Baillon et al., 1999). A *sodB* mutant was used as a control for menadione stress because SodB is the main enzyme that reduces superoxides (Palyada et al., 2009). The 11168H *sodB* mutant was highly sensitive to menadione exposure.

### Mutation of *rrpA* and *rrpB* Results in Reduced Growth Under Aerobic Stress Conditions

**FIGURE 6:**
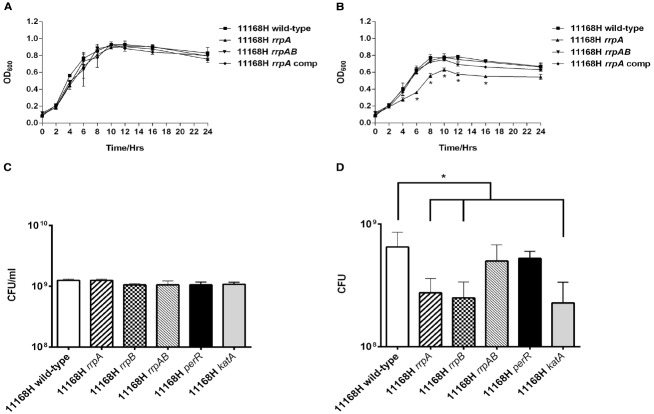
**Growth curves for *C. jejuni* 11168H wild-type strain, *rrpA* mutant, *rrpA* complement strain (*rrpA* comp) and *rrpAB* mutant grown under either microaerobic (A) or aerobic conditions (B) at 37°C (with shaking at 75 rpm) in Brucella broth with bacterial growth assessed by recording the OD_600_ of the culture at different time points.** Bacterial CFU were also assessed at 16 h under aerobic **(C)** and microaerobic **(D)** conditions. Asterisks denote a statistically significant difference (*p < 0.05).

The *rrpA* mutant was grown under microaerobic and aerobic conditions and growth assessed by measuring the OD_600_ at different time points. No significant differences were observed in the bacterial growth profile when comparing the wild-type strain, *rrpA* mutant, *rrpAB* mutant and the *rrpA* comp strain under microaerobic conditions (Figure 6A). Interestingly, under aerobic conditions, the *rrpA* mutant displayed a reduced growth profile compared to the 11168H wild-type strain, *rrpAB* mutant and the *rrpA* comp strains (Figure 6B). In addition to OD_600_, the CFUs were also assessed under microaerobic and aerobic conditions for a number of strains at 16 h. No differences in growth rates compared to the wild-type strain were observed under microaerobic growth conditions (Figure 6C). However, differences were observed under aerobic growth conditions. Both the *rrpA* and *rrpB* mutants exhibited reduced CFU compared to the wild-type strain (Figure 6D). These results indicate RrpA and RrpB may also play a role in regulating the aerobic (O_2_) stress response in *C. jejuni*.

### 11168H *rrpA* and *rrpB* Mutants Exhibit Enhanced Ability to Form Biofilms Under both Aerobic and Microaerobic Conditions

**FIGURE 7:**
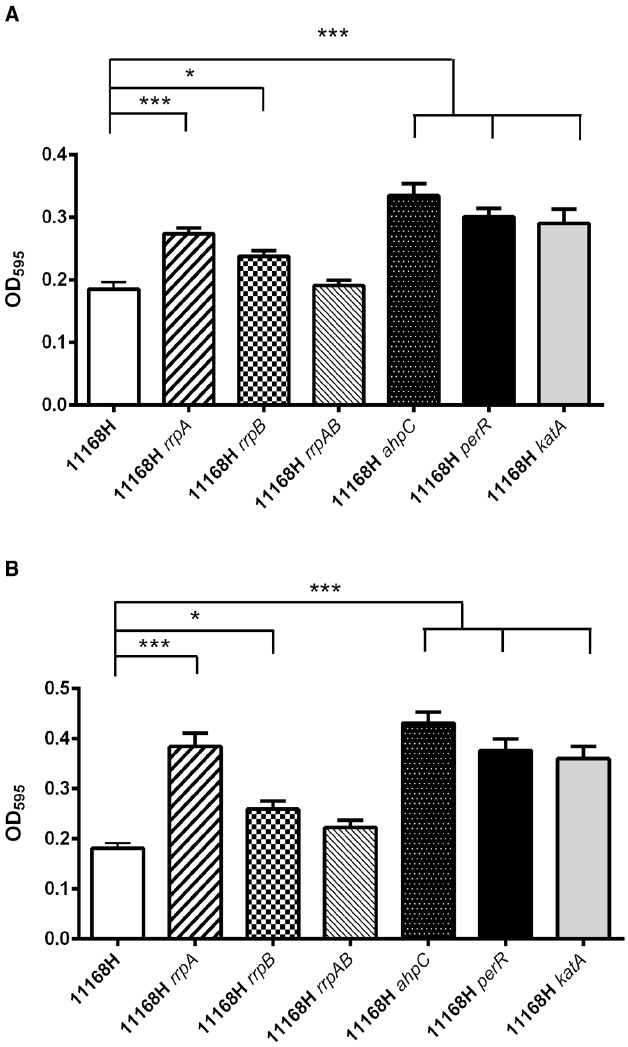
**Biofilm assays.** *C. jejuni* 11168H wild-type strain and mutants were grown for 72 h under aerobic **(A)** or microaerobic **(B)** growth conditions at 37°C without shaking, rinsed three times with PBS, followed by crystal violet staining. Asterisks denote a statistically significant difference (*p < 0.05; ***p < 0.001).

Studies have shown that *C. jejuni* can form biofilms (Joshua et al., 2006) and that this ability is an important factor in the ability of *C. jejuni* to survive in the ambient environment. It has previously been demonstrated that biofilm formation increases under aerobic stress conditions (Reuter et al., 2010). Both the 11168H *rrpA* and *rrpB* mutants exhibit an enhanced ability to form biofilms under both aerobic and microaerobic conditions after 72 h (Figure 7). However, the *rrpAB* mutant formed biofilms at a similar rate to the wild-type strain (Figure 7). As controls, biofilm assays were also performed on the *ahpC*, *perR*, and *katA* mutants, which displayed a similar high level of biofilm formation compared the wild-type strain after 72 h.

### 11168H *rrpA* Mutant Exhibits Reduced Cytotoxicity in the *Galleria mellonella* Larvae Model of Infection

**FIGURE 8:**
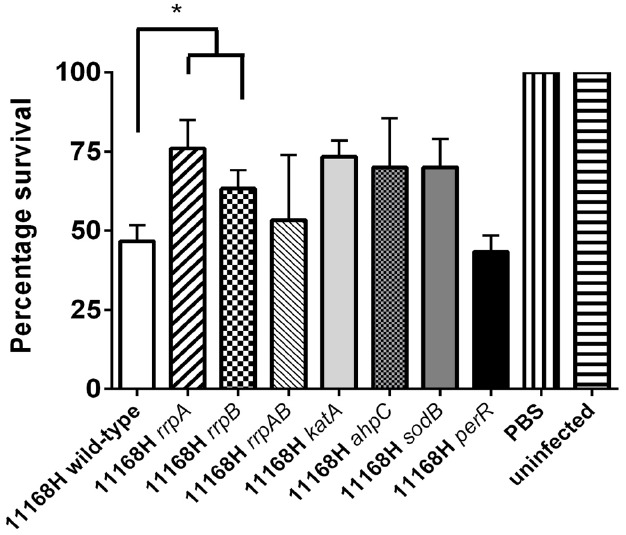
***Galleria mellonella* infection model.** *G. mellonella* larvae were injected with approximately 10^6^ *C. jejuni*. Larvae were incubated at 37°C with survival detailed at 24 h. Brucella broth and no injection controls were used. For each experiment, 10 *G. mellonella* larvae were infected and experiments were repeated in triplicate. Asterisks denote a statistically significant difference (*p < 0.05).

*Galleria mellonella* larvae are a convenient model to investigate the virulence of *C. jejuni* (Champion et al., 2009, 2010). The 11168H *rrpB* mutant has previously been shown to exhibit reduced cytotoxicity to *G. mellonella* larvae (Gundogdu et al., 2011). The 11168H *rrpA* mutant also exhibited reduced cytotoxicity to *G. mellonella* larvae compared to the wild-type strain (Figure 8). Both the *rrpAB* and *perR* mutants exhibited wild-type levels of cytotoxicity, whilst the *katA*, *sodB*, and *ahpC* mutants all exhibited reduced levels of cytotoxicity compared to the wild-type strain.

## Discussion

*Campylobacter jejuni* will be exposed to ROS during colonization or infection of a host, during survival in the environment and during the course of normal bacterial metabolism. An important question is how this widely dispersed and highly prevalent yet microaerophilic human pathogen is able to survive in the aerobic environment. *C. jejuni* has different mechanisms for opposing the effects of ROS and the control of *C. jejuni* oxidative stress responses is composite, involving multiple inter-linked levels of regulation (Atack and Kelly, 2009). The re-annotation of the *C. jejuni* NCTC11168 genome sequence (Gundogdu et al., 2007) identified both RrpA and RrpB as putative MarR-type transcriptional regulators and RrpB was previously shown to be involved in the *C. jejuni* oxidative and aerobic (O_2_) stress responses (Gundogdu et al., 2011). The data presented in this study demonstrates that RrpA as well as RrpB plays a role in the *C. jejuni* oxidative and aerobic (O_2_) stress responses, more specifically hydrogen peroxide stress response and the regulation of KatA expression.

A complex network of regulatory proteins is involved in controlling the *C. jejuni* oxidative stress response dependent on both the oxidant and other physiological conditions (Atack and Kelly, 2009). This control is multi-factorial with different regulatory proteins acting as repressors or activators and many regulators appearing to affect the expression of other regulatory proteins. Analysis of *C. jejuni* NCTC11168 gene expression changes induced following exposure to H_2_O_2_, cumene hydroperoxide or menadione identified three distinct transcriptional responses to these different oxidative stresses (Palyada et al., 2009). KatA, AhpC and SodB are particularly important in protecting *C. jejuni* against the oxidative damage induced by H_2_O_2_, cumene hydroperoxide and menadione respectively. Twelve genes were identified with a similar transcriptional profile to all three oxidative stresses, whilst 8, 11, and 12 genes were expressed differentially by H_2_O_2_, cumene hydroperoxide or menadione stresses respectively (Palyada et al., 2009). SodB expression was increased in response to menadione, demonstrating an important role in breaking down this compound (Palyada et al., 2009). The 12 genes associated with the response to all three oxidant stimulons are all regulated by PerR, indicating that PerR plays a role in responding to H_2_O_2_, cumene hydroperoxide and menadione induced oxidative stress (Palyada et al., 2009). A NCTC11168 *perR* mutant was shown to be more resistant to both H_2_O_2_ and cumene hydroperoxide oxidative stresses compared to the wild-type strain, yet was more sensitive to menadione oxidative stress (Palyada et al., 2009). PerR negatively regulates both *katA* and *ahpC* expression in an iron-dependent manner and a NCTC11168 *perR* mutant, that constitutively expressed both *katA* and *ahpC*, was hyper-resistant to oxidative stress (van Vliet et al., 1999). In this study, the 11168H *perR* mutant was shown to be resistant to exposure to both 50 and 100 mM H_2_O_2_ for 15 min, levels of oxidative stress that the wild-type strain was unable to survive. This hyper-resistance to oxidative stress of *perR* mutants was shown to be in part due to levels of KatA expression almost a 100-fold higher than that of the wild-type strain. The OmpR-type response regulator CosR has been shown to negatively regulate the expression of oxidative stress response proteins including SodB and to positively regulate AhpC (Hwang et al., 2011, 2012). Knockdown of CosR resulted in bacteria more resistant to oxidative stress, whilst bacteria overexpressing CosR were more sensitive to oxidative stress (Hwang et al., 2011). In addition, CosR expression was significantly reduced in the presence of paraquat (a superoxide generator), but not by H_2_O_2_ (Hwang et al., 2011). CosR also positively controls *katA* transcription and catalase activity by direct interactions with the *katA* promoter (Hwang et al., 2012).

A 11168H *rrpA* mutant displayed increased sensitivity to H_2_O_2_ (25 mM) compared to the wild-type strain, whilst neither the wild-type strain, *rrpA* nor *rrpB* mutants survived exposure to higher concentrations of H_2_O_2_ (50 or 100 mM) (Figure 2A). In contrast a 11168H *rrpAB* double mutant exhibited increased resistance to these concentrations of H_2_O_2_ (Figure 3A), as did a 11168H *perR* mutant, in agreement with the previous report that a NCTC11168 *perR* mutant was hyper-resistant to oxidative stress (van Vliet et al., 1999). The data from catalase activity assays suggests that the increased sensitivity to H_2_O_2_ of the *rrpA* and *rrpB* mutants is due to a decrease in KatA activity compared to the wild-type strain (Figure 2B), whilst the *rrpAB* mutant displays levels of KatA activity slightly higher (but not significantly so) than the wild-type strain (Figure 3B). Mutation of either *rrpA* or *rrpB* results in a reduction in the level of *katA* expression, whilst mutation of both *rrpA* and *rrpB* results in an increase in *katA* expression compared to the wild-type strain. However, the increase in *katA* expression in the *rrpAB* mutant was not significant, indicating the role of additional factors impacting the survival of the *rrpAB* mutant to H_2_O_2_. *C. jejuni* possesses a large repertoire of enzymes including a catalase (KatA), an alkyl hydroperoxide reductase (AhpC) and a superoxide dismutase (SodB) that allow the bacterium to tolerate and survive ROS (Atack and Kelly, 2009). KatA, AhpC, and SodB are particularly important in protecting *C. jejuni* against the oxidative damage induced by H_2_O_2_, cumene hydroperoxide and menadione respectively. In contrast to the results observed for H_2_O_2_ stress assays, both the *rrpA* and *rrpB* mutants displayed similar levels of sensitivity to either cumene hydroperoxide or menadione stress as the wild-type strain (Figures 5A,B). The *rrpAB* mutant displayed slightly increased resistance to cumene hydroperoxide and menadione stress compared to the wild-type strain (data not shown). This indicates that RrpA and RrpB are specifically involved in the hydrogen peroxide stress response and regulating expression of KatA, hence the designation of gene name based upon this distinction. This is supported by microarray data that showed that RrpB appears to positively regulate *katA* expression (Gundogdu et al., 2011). Additionally, in this study we have shown that RrpA binds not only upstream of itself indicating a classical MarR-type autoregulation (Figure 1A), but also binding upstream of *katA* and this provides evidence that RrpA directly influences the amount of catalase produced (Figure 1B). This gives a possible explanation as to why the *rrpA* mutant gave a reduced activity in the catalase activity assay. The binding capability of RrpA upstream of *katA* would suggest that RrpA is acting as an activator for *katA* and that mutation of *rrpA* reduces the amount of *katA* expressed and hence less catalase is produced in response to H_2_O_2_ stress.

The *rrpA*, *rrpB*, and *katA* mutants all exhibit reduced growth under aerobic conditions compared to the wild-type strain, suggesting that a reduction in KatA expression adversely affects the ability of *C. jejuni* to survive exposure to the aerobic (O_2_) stress under atmospheric conditions (Figures 6B,D). However, the *rrpAB* mutant exhibits wild-type levels of growth under aerobic conditions, in keeping with the similar levels of KatA expression. *C. jejuni* forms biofilms (Joshua et al., 2006; Gundogdu et al., 2011) which may be an important factor in the survival of *C. jejuni* in the environment. Biofilm formation has been linked to responses to both oxidative and aerobic (O_2_) stress and *C. jejuni* NCTC 11168 biofilm formation has been shown to occur more rapidly under aerobic conditions (Reuter et al., 2010). However, biofilm levels were also shown to be equivalent under aerobic or microaerobic conditions after 3 days of incubation (Reuter et al., 2010), in agreement with the data for 11168H presented here. Both the *rrpA* and *rrpB* mutants formed biofilms at a significantly higher rate compared to the wild-type strain under both aerobic and microaerobic conditions after 3 days (Figures 7A,B). The *ahpC*, *perR*, and *katA* mutants also formed biofilms at a significantly higher rate, whilst the *rrpAB* mutant formed biofilms at wild-type levels. Previously, a *C. jejuni ahpC* mutant was shown to increase biofilm formation, whilst a *perR* mutant was shown to reduce biofilm formation (Oh and Jeon, 2014). This data was obtained after 24 and 48 h in MH broth at 42°C without shaking under microaerobic conditions, whilst the data in this study was obtained after 72 h in MH broth at 37°C without shaking under both aerobic and microaerobic conditions. Such differences in the experimental conditions may account for these different observations, particularly as the levels of biofilm formation observed in this study were lower than those in the earlier study. There are considerable differences reported in the literature on the ability of *C. jejuni* to produce biofilms, often depending on the media used, whether shaking or stationary cultures were used, whether glass or plastic containers were used and the length of time the assays were performed for. Mutation of *ahpC* was associated with an increase in the accumulation of ROS within the cytoplasm (Oh and Jeon, 2014). Further studies are required to identify the type of ROS responsible for inducing *C. jejuni* biofilm formation and why this is increased under aerobic conditions.

*Galleria mellonella* larvae possess specialized phagocytic cells, termed haemocytes. Similar to mammalian phagocytic cells, haemocytes are capable of internalizing bacterial pathogens and generating bactericidal compounds (Bergin et al., 2005; Champion et al., 2009). There are many types of haemocytes identified in insects such as *G. mellonella* with plasmatocytes and granulocytes the most common (Boman and Hultmark, 1987). The production of ROS has been identified in haemocytes with both H_2_O_2_ and oxygen radicals identified in *G. mellonella* plasmatocytes (Slepneva et al., 1999). Infection of *G. mellonella* larvae with *rrpA* or *rrpB* mutants resulted in increased survival of larvae compared to the wild-type strain, indicating that the increased sensitivity of these mutants to H_2_O_2_ results in lower levels of bacterial survival and thus reduced cytotoxicity to the *G. mellonella* larvae (Figure 8). This is supported by the fact that the *katA*, *sodB*, and *ahpC* mutants also all exhibit reduced cytotoxicity. However, no increase in survival of *G. mellonella* larvae was observed after infection with either the *rrpAB* or *perR* mutants, probably as these mutants are able to survive as well as the wild-type strain within the larvae.

Mutation of *perR*, knockdown of CosR expression or mutation of both *rrpA* and *rrpB* all result in increased resistance of *C. jejuni* to oxidative stress, indicating that all four regulators act to repress KatA expression in the absence of peroxide stress (van Vliet et al., 1999; Palyada et al., 2009; Gundogdu et al., 2011; Hwang et al., 2011, 2012). However, the single mutation of either *rrpA* or *rrpB* results in a reduction in KatA activity and decreased resistance to peroxide stress, suggesting that the absence of either RrpA or RrpB negatively impacts the ability of *C. jejuni* to express KatA in response to peroxide stress. However, the mutation of both *rrpA* and *rrpB* results in no significant change in KatA activity but increased resistance to peroxide stress. This is the most difficult phenotype to explain, suggesting that in the absence of both RrpA and RrpB, catalase expression is not significantly affected, possibly via compensatory regulation involving PerR and CosR. The fact that the *rrpAB* double mutant exhibits increased resistance to both cumene hydroperoxide and menadione stress suggests a more complicated regulation of the *C. jeuni* oxidative stress responses. Further studies are required to understand how RrpA and RrpB regulate KatA expression and interact with PerR and CosR. The crystal structure of an *E. coli* MarR protein, determined at a resolution of 2.3 A, indicates that MarR forms dimers with each subunit containing a winged-helix DNA binding motif (Alekshun et al., 2001). If RrpA and RrpB form only homo-dimers, this would suggest that both dimer complexes are required for the efficient regulation of KatA expression and the absence of either dimer complex results in the reduced ability of *C. jejuni* to express KatA. However, in the absence of both RrpA and RrpB dimer complexes, KatA expression is not significantly affected. Further studies are required to clarify the exact roles of RrpA and RrpB in the *C. jejuni* responses to both oxidative and aerobic (O_2_) stress and how this enhances bacterial survival both *in vivo* and in the environment.

### Conflict of Interest Statement

The authors declare that the research was conducted in the absence of any commercial or financial relationships that could be construed as a potential conflict of interest.
